# Editorial: Urban health and planning in the 21st Century: bridging across the formal and informal using an eco-social lens

**DOI:** 10.3389/fpubh.2024.1367882

**Published:** 2024-03-13

**Authors:** Ritu Priya, Sayan Das, Unnikrishnan Payyappallimana, John Porter, Mathew George, Carolyn Stephens, Jose Siri

**Affiliations:** ^1^Centre of Social Medicine and Community Health, Jawaharlal Nehru University, New Delhi, India; ^2^Center for Community Health, Clinical Research and Education, Trans Discipilinary University, Bangalore, India; ^3^Faculty of Public Health and Policy, London School of Hygiene and Tropical Medicine, University of London, London, United Kingdom; ^4^Department of Public Health and Community Medicine, Central University of Kerala, Kasaragod, Kerala, India; ^5^London School of Hygiene and Tropical Medicine, University of London, London, United Kingdom; ^6^World Health Organization, Geneva, Switzerland

**Keywords:** eco-social perspective, urban informality, urban health care, urban wellbeing, pathways to bridging formal-informal, sustainable urbanization, urban governance, rural-urban continuum

In today's rapidly urbanizing world, the issues surrounding the nature of urbanization, urban habitats and urban policies have become critical to ensure the collective health and wellbeing of people, animals, and the planet. Environmental degradation and climate change, emerging infectious diseases, rising non-communicable diseases at younger ages, increasing urban population density, and intensifying inequalities in access to healthcare, among others, pose serious challenges today, demanding a creative rethink of urban health and its determinants.

Urban health and wellbeing emerge from the dynamic relationship shared between urban dwellers and their living environments, shaped by their differential capabilities to meet habitat, healthcare and other needs in light of planned urban development. Urban living, involving health-related practices, livelihoods or quotidian amenities and routines, typically bridges the formal and informal divide present in policy. The complementarities and contradictions shaping the complexity of urban health need multimodal explorations into not just the urban health system but also urban design, planning and management for sustainable urbanization. The break in the relationship between public health and urban planning as twin approaches to address the same set of problems needs to be remedied.

In urban planning, there is increasing recognition of the value of understanding the “informal” as a ubiquitous presence that merits incorporation into formal planned interventions (1). In public health, however, this is not yet as pervasive an idea, despite the well studied presence of “the folk” in health-related practices, and even less so when thinking about urban health (2).

The studies included under this Research Topic bring attention to the phenomenon of the “informal” spanning a wide range of issues from examining determinants of urban health for rural-to-urban migrants to indicating potential pathways that attempt to bridge across the formal and informal, to some methodological approaches for assessing such interventions. Zhao et al., for instance, interrogate how factors such as information transmission, health habits, social capital, and cultural identity, in addition to access to quality healthcare, shape the migrants' urban health-related experience and practice. The impacts of urbanization on health, however, can unfold in seemingly different ways as two studies in this collection found. Luo and Wang, analyzing a baseline survey of chronic diseases from 2011 to 2021 in southeastern China, observed a statistically significant relationship between urban living and the prevalence of type 2 diabetes, hyperlipidemia, and hypertension which, however, diminished with improvement in the healthcare system. Among the mediators, psychological distress had the highest positive coefficient for these diseases. Curiously, the study by Li et al., examining potential gender differences in the association of urbanization with psychological stress among Chinese adults, found that women in the most urbanized communities were likely to have lower levels of psychological stress than those in the low urbanized communities. Changing gender roles in the urban context, the authors explain, may have eased women out of stressful family environments while the crowded urban areas allowed greater access to modern markets, mental health professionals as well as neighborhood social support.

Social support in the urban context, especially for the underprivileged, often involves a complex interplay of different actors and networks. The absence of adequate and quality formal services in informal settlements is often filled by the urban poor through informal means in a show of resilience. That however cannot be an excuse for inaction by the formal system. The study by Chumo et al., conducted across two informal settlements in Nairobi, found that a complementary role of formal and informal actors including their respective networks is essential to supporting vulnerable populations. Chumo et al.'s other paper on Community Advisory Committees in informal settlements of Africa highlights the significance of social participatory mechanisms for advancing community health needs and priorities in an environment otherwise characterized by “a combination of patronage and neglect, insecurity and inequity.”

Urbanization creates both opportunities and exclusions. Issues of equity critically determine the varied experiences across diverse urban populations (3). While there have been attempts at mapping urban inequities, whether in terms of socio-economic status or health-seeking, the study by Lowensen et al. examines the contribution of diverse health-promoting urban initiatives to different dimensions of health equity in East and Southern Africa. Most initiatives in low-income communities prioritized social determinants of health, such as water-sanitation-waste management, energy, land, biodiversity, urban agriculture and food security as precursors to urban health and wellbeing. Across these initiatives participation of different social groups and recognition of their differential needs was better represented than distributional, structural and intergenerational equity. Gao et al. also explored equity in access to public green spaces across American neighborhoods during the COVID-19 pandemic, finding that it worsened particularly for the marginalized groups.

While all these papers used innovative methods to study multi-dimensional issues in relation to urban living, two studies specifically examined methodological approaches. Eaton et al. developed a tool by using an environmental economics approach to estimate the health impacts of environmental change. Given the challenges involved in synthesizing a wide range of evidence for the exercise, the authors do however caution that expert interpretation and contextual understanding are critical. The other study by Nel et al. explicated the use of Broad Brush Surveys for a quick qualitative assessment of the formal-informal processes involved in water and sanitation services in the context of rapid urbanization. Both tools hold promise for assessing and estimating the impacts of urbanization.

We hope these papers will move the public health and urban planning discussions forward in recognizing the value of bridging the formal-informal dimensions of health and healthcare in developing urban systems with the goal of health for all being a central tenet. There are multiple global and regional policy-oriented networks including that of WHO and UNESCO that are involved in identifying good practices; promoting multi-stakeholder city-to-city learning exchanges as well as developing institutional and evidence-based policy frameworks for improved governance (4). We do hope that the significance of bridging the formal and informal will be of value to these processes as well.

## Author contributions

RP: Conceptualization, Writing—original draft, Writing—review & editing. SD: Conceptualization, Writing—original draft, Writing—review & editing. UP: Writing—original draft, Writing—review & editing. JP: Writing—original draft, Writing—review & editing. MG: Writing—original draft, Writing—review & editing. CS: Writing—original draft, Writing—review & editing. JS: Writing—original draft, Writing—review & editing.
